# Identification of lactylation related model to predict prognostic, tumor infiltrating immunocytes and response of immunotherapy in gastric cancer

**DOI:** 10.3389/fimmu.2023.1149989

**Published:** 2023-03-03

**Authors:** Hao Yang, Xiaoming Zou, Shifeng Yang, Ange Zhang, Nana Li, Zhen Ma

**Affiliations:** Department of General Surgery, The Second Affiliated Hospital of Harbin Medical University, Harbin, Heilongjiang, China

**Keywords:** lactylation, prognosis, infiltrating immunocytes, immuntherapy, gastric cancer

## Abstract

**Background:**

The epigenetic regulatory chemical lactate is a product of glycolysis. It can regulate gene expression through histone lactylation, thereby promoting tumor proliferation, metastasis, and immunosuppression.

**Methods:**

In this study, a lactylation-related model for gastric cancer (GC) was constructed, and its relationships to prognosis, immune cell infiltration, and immunotherapy were investigated. By contrasting normal tissues and tumor tissues, four lactylation-related pathways that were substantially expressed in GC tissues were found in the GSEA database. Six lactylation-related genes were screened for bioinformatic analysis. The GC data sets from the TCGA and GEO databases were downloaded and integrated to perform cluster analysis, and the lactylation related model was constructed by secondary clustering.

**Results:**

The fingding demonstrated that the lactylation score has a strong correlation with the overall survival rate from GC and the progression of GC. Mechanistic experiments showed that abundant immune cell infiltration (macrophages showed the highest degree of infiltration) and increased genetic instability are traits of high lactylation scores. Immune checkpoint inhibitors (ICIs) demonstrated a reduced response rate in GC with high lactylation scores. At the same time, tumors with high lactylation scores had high Tumor Immune Dysfunction and Exclusion scores, which means that they had a higher risk of immune evasion and dysfunction.

**Discussion:**

These findings indicate that the lactylation score can be used to predict the malignant progression and immune evasion of GC. This model also can guide the treatment response to ICIs of GC. The constructed model of the lactate gene is also expected to become a potential therapeutic target for GC and diagnostic marker.

## Introduction

One of the most prevalent malignant tumors in the world is gastric cancer (GC), which poses a severe threat to human health (1). The evolution of GC is a complicated pathological process involving a number of variables and phases, which is the result of the interaction between dietary factors, host genes, *Helicobacter pylori* infection, and environmental factors. Due to the atypical symptoms of early-stage GC, majority of cases are already advanced when they are diagnosed. GC patients typically have a poor prognosis, a high risk of local recurrence, and distant metastasis (2–4). As GC research has advanced, it has been found that GC is not caused only by specific gene mutations, but cellular metabolic dysfunction is also a key marker of GC progression (5–8). There is mounting evidence that tumor metabolism is crucial to the initiation and development of malignancies as well as affecting immune cells through the release of metabolites (such as lactate and arginine). There is metabolic competition in the tumor environment as a outcome of this energetic transition between tumors and immunocytes, which limits effective supply of nutrients and leads to microenvironmental acidosis, thereby hindering the function of immune cells (9).

Lactate was once considered only a metabolite of glycolysis and the final product of the Warburg effect. A rising number of research have revealed that lactate is not just an essential energy source., signaling molecule, and immunomodulatory molecule but can also control body’s metabolism, immunological response, and intercellular communication (10). When tumor cells undergo abnormal glycolysis, they continue to intake a lot of glucose and make a lot of lactate even when there is plenty of oxygen available to them (11). Lactate accumulates in the cells and is exported to the extracellular environment by activating the transport proteins on the cell membrane, eventually forming the acidic tumor microenvironment. In addition to providing the energy required by cells as a fuel substrate, lactate can be utilized as a precursor material to modify histone lysines through lactylation (12). Histone lactylation is a crucial mechanism through which lactate performs its duties and takes part in crucial biological processes, such as glycolysis-related cellular functions (13), macrophage polarization (14), neurodevelopment (15), and regulation of tumor proliferation (16).

Although the lactylation modification has received extensive attention, the relevant articles are still limited. In particular, there are few articles on lactylation modification in GC. This detailed investigation of lactylation-related gene expression and relevance in GC was carried out by us. First, by comparing normal gastric tissues and GC tissues, four lactylation-related pathways with significantly elevated gene expression in GC tissues were identified by GSEA. We hypothesize that these pathways directly or indirectly contribute to the development of GC, leading to a poorer prognosis. After differential analysis and univariate Cox analysis of the above pathways, we obtained six prognostic lactylation genes. By secondary clustering based on these six lactation-related genes, we constructed a model (“lactylation score”) that classified as potential screening molecules for GC, which helped to discover various immunocellular infiltration and genetic instability patterns.

**Figure 1:**
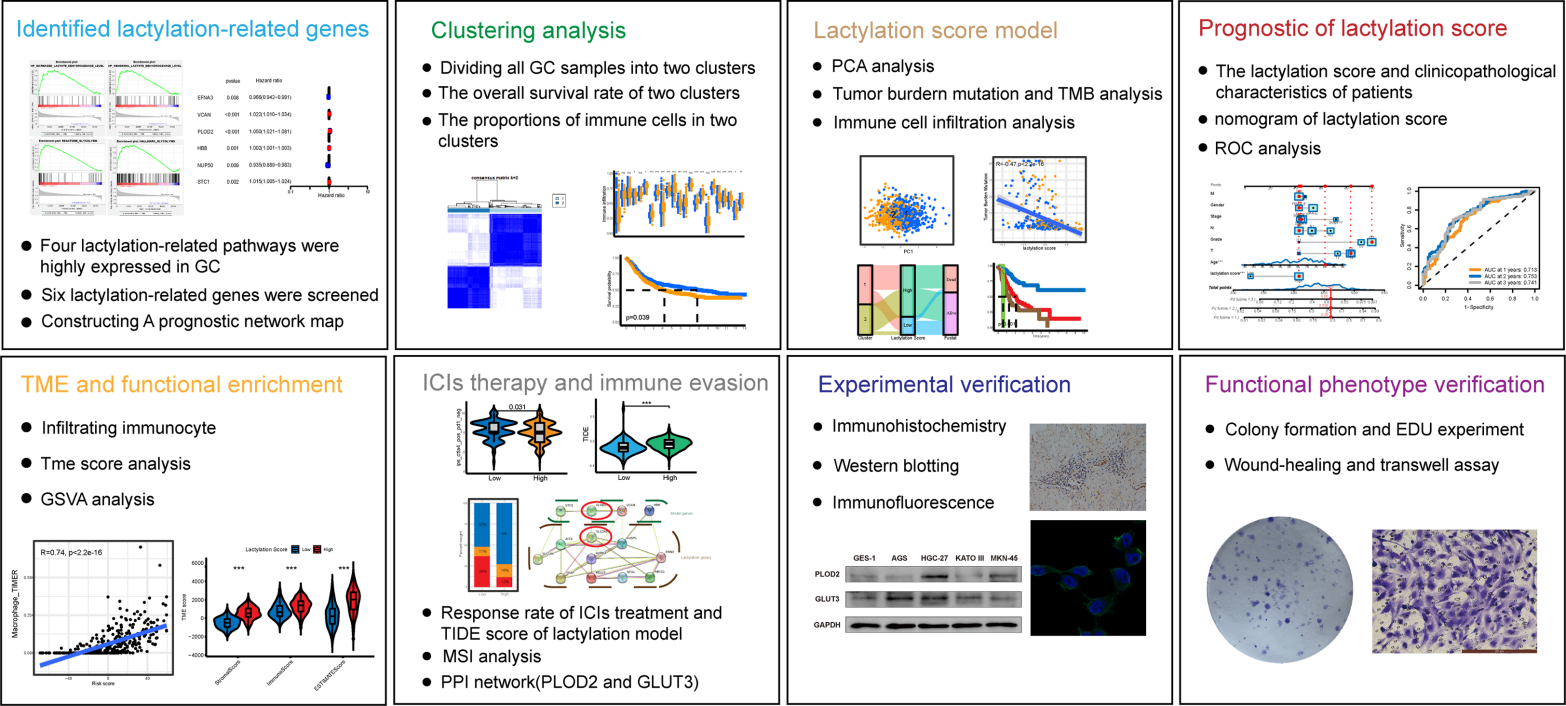
Flowchart of the study.

Through further analysis, the results revealed that GC patients with high lactylation scores possessed greater potential for immune evasion and lower rates of immunotherapy response, which also means that the lactylation score could become a method for forecasting patients’ reactions to immune checkpoint inhibitor (ICI) therapy. The scoring model’s PPI network was built, and the hub gene PLOD2 and its downstream lactylation target gene GLUT3 were chosen for experimental validation. Both GC cell lines and GC tissues have significantly higher levels of PLOD2 and GLUT3 expression. After treating GC cell lines with lactate dehydrogenase inhibitors, their expression was decreased, demonstrating the strong relationship between lactylation and the expression of these two genes. Flowchart of this study shows in **Figure 1**.

## Materials and methods

### Data retrieval and processing

Through the GSEA database (https://www.gsea-msigdb.org, December 2021), lactylation-related pathways and their genes were downloaded. Through the TCGA database (https://portal.gdc.cancer.gov/, December 2021), we obtained the raw mRNA matrix data in fragments per kilobase million (FPKM) format and copy number data of GC tissues. From the TCGA database, we also obtained relevant clinical data of the GC patients. Baseline characteristics of patients was summarized in **Table 1**. We downloaded the GSE84437 dataset from the Gene Expression Omnibus (GEO) database (https://www.ncbi.nlm.nih.gov/geo/, December 2021) to obtain the mRNA matrix and the clinical data of GC patients. The batch effect was removed using ComBat function of the SVA package in R for subsequent analysis. The STRING website (https://string-db.org/cgi/input.pl, December 2021) was used to construct the PPI network of lactylation-related genes.

**Table 1:** Baseline characteristics of patients from TCGA and GEO database.

Clinical features	Total patients (803)		TCGA (377)		GSE84437 (426)	
	Number	Percentage	Number	Percentage	Number	Percentage
		(%)		(%)		(%)
Age						
<=65	456	56.79	176	46.68	280	65.73
>65	347	43.21	201	53.32	146	34.27
Gender						
Female	265	33	130	34.48	135	31.69
Male	538	67	247	65.52	291	68.31
Fustat						
Alive	451	56.16	227	60.21	224	55.58
Dead	352	43.84	150	39.79	202	47.42
Grade						
1-2	135	16.81	135	35.81	0	0
3	242	30.14	242	64.19	0	0
Unkown	426	53.05	0	0	426	100
Stage T						
1-2	147	18.31	98	25.99	49	11.5
3-4	656	81.69	279	74.01	377	88.5
Stage N						
N0	196	24.41	116	30.77	80	18.78
N1-3	607	75.59	261	69.23	346	81.22

### Screening of prognostic lactylation-related genes in GC

By performing differential analysis and univariate Cox analysis of lactylation-related pathways in all GC samples, we obtained six prognostic lactylation-related genes. The copy number variation frequency of lactylation-related genes was calculated from the increase and decrease in gene copy numbers in GC samples from the TCGA database. The number of gene mutations in GC samples from the TCGA database was calculated to draw a waterfall plot. The “RCircos” package of the R language was used to plot the gene copy number circle diagram. Through Cox analysis and coexpression analysis, the prognostic network of lactylation-related genes was plotted. We used the Kaplan–Meier method to calculate the survival curves of GC patients and plotted them using the “survminer” package (17). The clinical data and lactylation scores of all GC patients were analyzed to calculate survival time, survival status, and risk division and to construct nomograms and receiver operating characteristic (ROC) curves.

### Cluster analysis

We categorized the GC cohort by “ConsensusClusterPlus” package to determine whether the expression of genes related to lactylation was connected with GC. The intragroup correlation is strong and the intergroup correlation is modest for k=2. Using heatmaps, we connected the amounts of lactylation-related gene expression in several types of GC with patient clinical information. We measured the immune cell expression in various GC types using the ssGSEA technique, and then plotted the data using box plots. Through the GSEA website (https://www.gsea-msigdb.org, December 2021), the files of the Gene Ontology (GO) and Kyoto Encyclopedia of Genes and Genomes (KEGG) pathways were downloaded, and the functional pathways enriched in GC types were plotted as a heatmap using the “GSEABase” and “GSVA” packages of R.

### Construction of the lactylation score model

We developed an lactylation score scheme to quatify the lactylation modification level of individual patients by using PCA. Specifically,the overlapping genes(3112) identified from two clusters of GC were selected and employed to perform prognostic analysis for each gene using a univariate COX regression model. The genes with a significant prognostic were extracted for further feature selection by using recursive feature elimination(RFE) with random forest and the 10-fold cross-validation method in the caret package. We then curated the expression profile of the final determined genes to perform PCA analysis, and principal components 1 and 2 were extracted and served as the signature score. We then adopted a formula similar to previous studies to define the lactylation score: lactylation score =∑(PC1i+PC2i).

We performed correlation analysis between lactylation score and immune cells present in the tissue using the ssGSEA algorithm. With the “ggpubr” and “reshape2” packages of R, we analyzed the relationship between clustering classification, lactylation score, and tumor mutation burden. With the “survival” package and the “survminer” package, we conducted joint survival analysis of the high-tumor-mutation-load group, low-tumor-mutation-load group, high-lactylation-score group, and low-lactylation-score group. With the “plyr” package and the “ggpubr” package, we plotted the different clinicopathological features in the lactylation score group as bar graphs and box plots. From the Cancer Immunome Database (TCIA) (https://tcia.at/, December 2021), the scoring data of ICI treatment and MSI status for GC were downloaded. According to lactylation score, we analyzed the ICI treatments CTLA-4 and PD-1 for pancreatic cancer (18). All gastric cancer patients were divided into MSS, MSI-L and MSI-H groups, and lactylation scores among all groups were calculated. The Tumor Immune Dysfunction and Exclusion (TIDE) score, exclusion score, and dysfunction data of GC were downloaded from the TIDE database (http://tide.dfci.harvard.edu/, December 2021), and the immune evasion and immune dysfunction in high- and low-lactylation-score groups were analyzed. We used “pRRophetic” package of R to predict drug sensitivity of high- and low-lactylation score groups (p<0.001).

### Collection of tissue samples from GC patients

Tissue samples were collected from GC patients who underwent surgical resection at the Second Affiliated Hospital of Harbin Medical University. These GC patients had not received any other treatments, such as radiotherapy, chemotherapy, or biological treatment, before surgery. All specimens were histopathologically diagnosed by two pathologists according to the diagnostic criteria for GC. All patients provided informed consent.

### Cell culture and transfection

Normal gastric mucosal epithelial cells (GES-1) and GCcells (AGS, HGC-27, KATO3, MKN-45) were purchased from Procell Life Science & Technology (Wuhan, China), and the cells were cultured according to the manual instructions. A lactate dehydrogenase A (LDHA) inhibitor (GSK2837808A, MCE) was used to treat HGC cell lines. The HGC cell lines were cultured in six-well plates, and plasmids were transfected using Lipofectamine 3000 according to the instructions. The target sequences of the short hairpin RNA (shRNA) were as follows: PLOD2-RNAi (8407-1), caGCAAGTGTCCTTAAGTCAA; PLOD2-RNAi (8408-1), ggAAATGGACCCACCAAGATT; PLOD2-RNAi (8409-1), ctTTGCCGAAATGCTAGAGAA.

### Western blotting

WB assay was done in accordance with prior literature descriptions (19). We used RIPA buffer to extract the proteins needed for western blotting. The subsequent antibodies were utilized: PLOD2 (Proteintech, China), GLUT3 (Abcam, UK) and GAPDH (ImmunoWay, USA).

### Immunohistochemistry

The tissue samples were sectioned at 5 μm after being submerged in 4% paraformaldehyde for an entire night. Following an overnight incubation at 4°C with anti-PLOD2 and anti-SLC2A3 antibodies, tissue slices were treated with secondary antibodies conjugated to horseradish peroxidase (20).

### RNA extraction and RT-PCR

TRIzol was applied to extract cellular RNA, and a cDNA synthesis kit was applied to create single-stranded cDNA from the recovered cellular RNA (21). The FastStart Universal SYBR Green Master Mix’s instructions were followed to perform the qPCR analysis. A 10-µl reaction system was prepared.

### Colony formation assays

After 10 days of incubation, the GC cell lines were seeded in a six-well plate, and colony formation was apparent to the naked eye.

### Ethylenediurea experiment

EdU experiment was performed according to the previous literature (22, 23).

### Wound-healing assay

Cells were cultured in six-well plates until full confluence, then starved with serum-free medium. We scratched a 10-μl pipette tip across the plate, removing a line of cells. Under a microscope, pictures were obtained at 0, 12, and 24 hours to record the extent of wound healing (24).

### Transwell assays

The GC cell lines were inoculated into a Transwell chamber containing 200 µl of serum-free medium. Matrigel mix was coated on the upper chamber surface of the Transwell chamber to detect the invasion ability of the cells. When testing the cell migration ability, the bottom of the chamber does not need to be coated with Matrigel. Medium containing 10% FBS was added to the lower culture plate. After 24 h of incubation, the chamber was removed and stained with crystal violet for 30 min. Five randomly selected fields of view were photographed, and their cells were counted under a microscope.

### Data analysis

The GraphPad Prism 8.0 software was used to illustrate the results of the data analysis, which was carried out using the SPSS 18.0 program. In particular, the means of the two groups were compared using Student’s t tests, and one-way ANOVA analysis was used to determine the statistical significance of the means from multiple groups (>2). The one-way ANOVA analysis for the corrective test was followed by the *post hoc* testing (Tukey test).

## Results

### Expression of lactylation-related genes in GC

**Figure 2:**
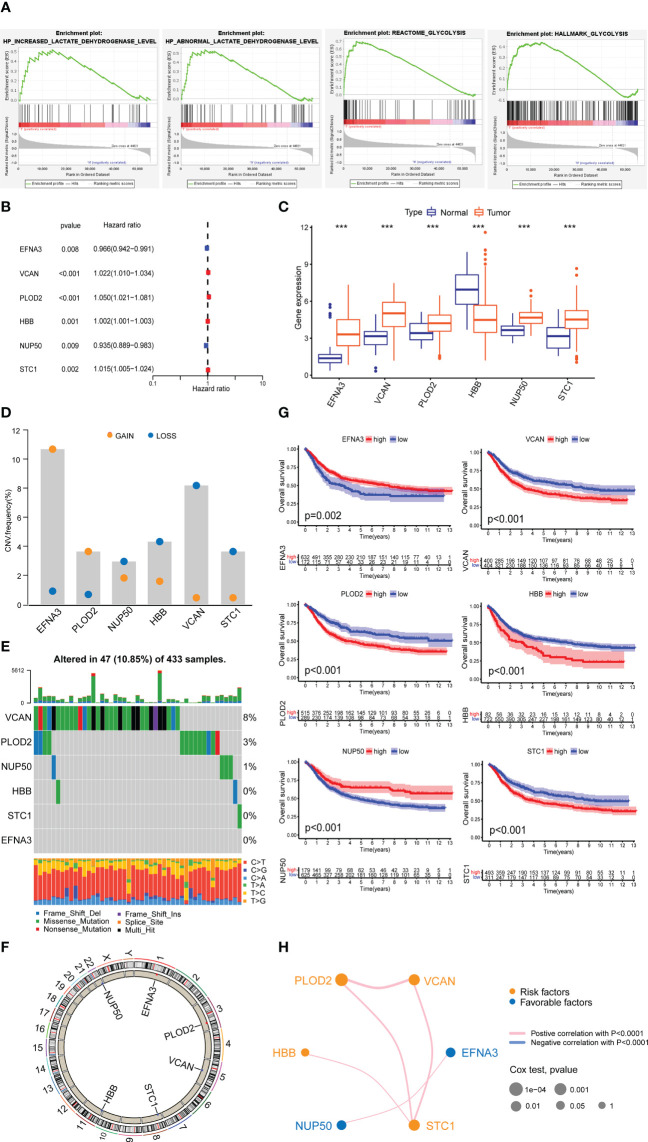
Expression of lactylation-related genes in GC. **(A)** Highly expressed GSEA pathway in GC tissues. **(B)** Univariate Cox analysis of lactylation-related genes. **(C)** Expression of lactylation-related genes in GC tissues and normal gastric tissues. ***p<0.001. **(D)** Diagram of the copy number frequencies of lactylation-related genes. **(E)** Waterfall plot of the mutation frequencies of lactylation-related genes. **(F)** Circle plot of the copy numbers of lactylation-related genes. **(G)** Overall survival rate of lactylation-related gene patient groups. **(H)** Prognostic network of lactylation-related genes.

Through the GSEA database, we identified four lactylation-related pathways that were significantly upregulated in GC tissues (**Figure 2A**). We speculate that these four lactylation-related pathways are directly linked to the incidence and evolvemetn of GC.

After differential and Cox analysis of the above lactylation pathways, we obtained six prognostic lactylation genes and plotted a forest diagram (**Figure 2B**). Box plot showed the expression of lactylation-related genes in normal gastric samples and GC samples (**Figure 2C**). Additionally, we examined the prevalence of copy number variation in target genes across all GC samples (**Figure 2D**). Except for *EFNA3* and *PLOD2*, which showed increased copy number, the other four genes all showed copy number reductions. We also plotted single-gene mutation frequency waterfall plots (**Figure 2E**) and gene copy number circle plots (**Figure 2F**) of the six lactylation-related genes.

Next, we combined the TCGA and GEO data to analyze the data of a total of 804 GC patients and performed Kaplan–Meier survival analysis based on lactylation-related gene groups (**Figure 2G**). The results showed that the survival curves of the six lactylation-related genes all had statistically significant differences. A prognostic network map was constructed through coexpression analysis (**Figure 2H**). It can be seen from the network map that *NUP50* and *EFNA3* were favorable factors, and the other four lactylation genes were risk factors. They regulate each other within a network, which can form a functional whole and together affect the progression of GC.

### Cluster analysis

**Figure 3:**
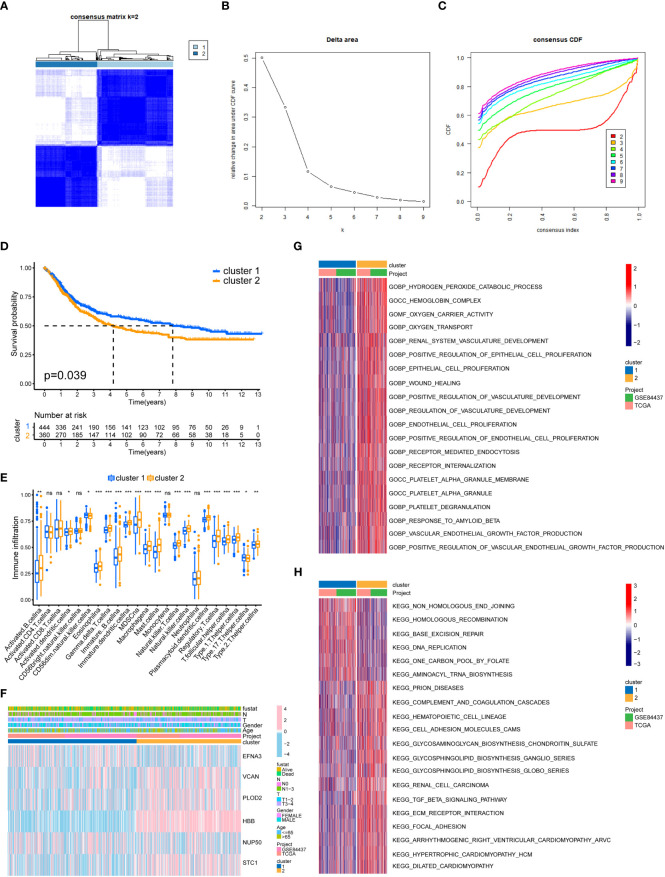
GC classification of lactylation-related genes. **(A)** Changes in the length and inclination of the cumulative distribution function (CDF) curve when k=2-9. **(B)** Area under the CDF curve when k=2-9. **(C)** GC samples were divided into two tumor clusters. **(D)** Kaplan–Meier survival curves of the two clusters. **(E)** Immune cell infiltration of the two clusters. *p<0.05; **p<0.01; ***p<0.001; ns, no signifificance. **(F)** Heatmap of GC classification, lactylation-related genes, and clinicopathological characteristics. **(G)** GO enrichment analysis of the two GC clusters. **(H)** KEGG enrichment analysis of the two clusters.

We separated all GC samples into two clusters using cluster analysis of lactylation-related genes (**Figures 3A–C**). Significant variations between the two clusters of GC were shown by the survival analysis (**Figure 3D**). Compared to cluster 1, cluster 2 had a much lower survival rate. We determined the ratios of 23 different immune cell types in the two clusters of GC using the ssGSEA method. 18 types of immunocytes were significantly different between the two clusters of GC (**Figure 3E**). We also plotted the clinicopathological characteristics and lactylation genes of GC samples into a heatmap (**Figure 3F**). Using the GSVA algorithm, we plotted the GO and KEGG pathways enriched in the two clusters of GC into a heatmap. The GO-enriched pathway in cluster 2 was mostly focused on angiogenesis and epithelial cell proliferation (**Figure 3G**), such as ENDOTHELIAL_CELL_PROLIFERATION and REGULATION_OF_VASCULATURE_DEVELOPMENT. The KEGG-enriched pathways in cluster 2 was mostly focused on metastasis-related pathways (**Figure 3H**), such as FOCAL_ADHENSION, and TGF_BETA_SIGNALING_PATHWAY.

### Constructing lactylation score model

**Figure 4:**
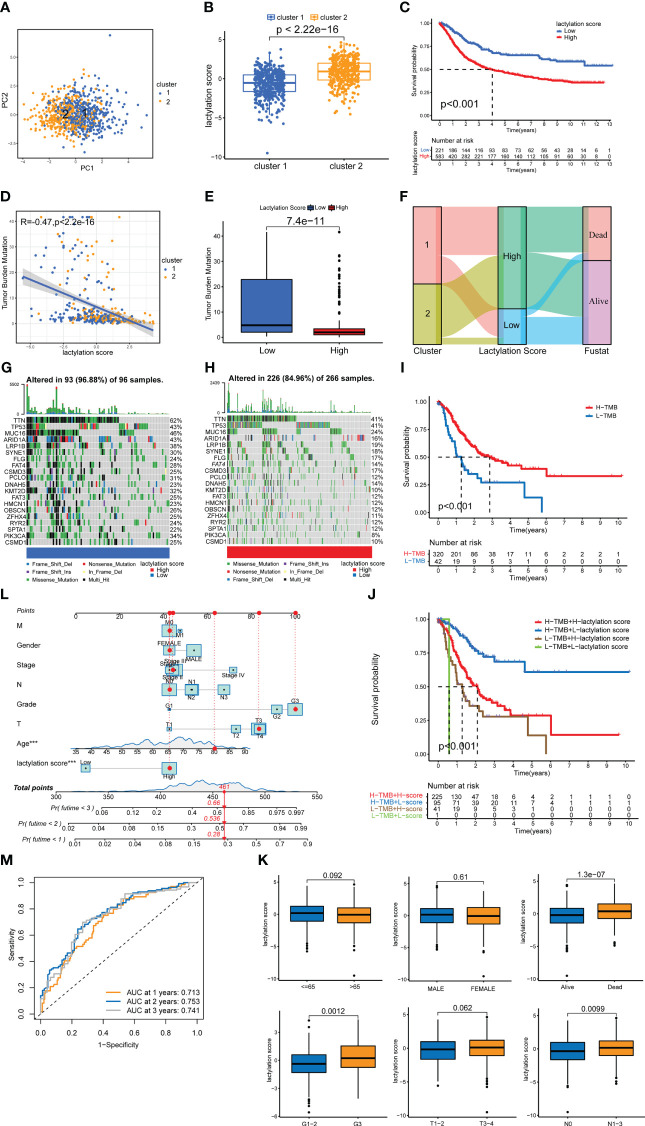
lactylation score model. **(A)** PCA of lactylation-related genes. **(B)** Lactylation scores of GC types. **(C)** Kaplan–Meier survival curve of the high- and low-lactylation-score groups. **(D)** Spearman correlation analysis of the lactylation score and immune cells. **(E)** *UBQLN4* expression in the high- and low-lactylation-score groups. **(F)** Sankey plot of GC clusters, lactylation score, and patient survival. **(G)** Waterfall plot of mutation frequencies in the low-lactylation-score group. **(H)** Waterfall plot of mutation frequencies in the high-lactylation-score group. **(I)** Kaplan–Meier survival curve of the high-tumor-mutation-burden group and the low-tumor-mutation-burden group. **(J)** Joint survival analysis was performed in the high and low-tumor-mutation-burden groups and the high- and low-lactylation-score groups. **(K)** The age, sex, survival, grade, T stage, and N stage of patients in the high- and low-lactylation-score groups. **(L)** Nomogram of the lactylation score and clinical information. ***p<0.001 **(M)** AUC of the lactylation score model.

The results of PCA on all GC samples (**Figure 4A**) revealed that there was little overlap between the two GC clusters and that the components within each GC cluster were well correlated. **Figure 4B** demonstrates that cluster 2’s lactylation score was substantially greater than cluster 1’s. The survival rate of high lactylation score group was considerably lower than low lactylation score group (**Figure 4C**), demonstrating that the prognosis is worse the higher the lactylation score. The TMB analysis was also examined in both groups, and the findings revealed a negative correlation between the two (**Figures 4D, E**). We also constructed a Sankey plot of the cluster of GC, lactylation score, and patient survival. **Figure 4F** shows that cluster 2 GC had a high correlation with a high lactylation score, while cluster 1 was associated with a low lactylation score. While the most part of samples in the low-lactylation-score group were in the survival condition, the majority of the patients who died belonged to the high-lactylation-score group. We created a waterfall plot to compare the prevalence of single-gene mutations in two groups (**Figures 4G, H**). The low-lactylation-score group had a higher prevalence of single-gene mutations than the high-lactylation-score group. **Figure 4I** demonstrates that the high-TMB group’s survival rate was significantly greater than the low-TMB group’s. Additionally, we performed conjoint analysis between tumor mutation burdens groups and lactylation score groups (**Figure 4J**). The result revealed statistical differences, demonstrating that the prognosis of GC patients was influenced by both TMB and lactylation scores.

### Prognosis and clinicopathological characteristics in different lactylation score groups

Additionally, we examined the patients’ clinicopathological traits, as well as their lactylation score. Age, sex, and T stage showed no significantly different between two lactylation scores groups, as shown in **Figure 4K**. Additionally, a poor prognosis, a high tumor grade, and lymph node metastases were all closely related to a high lactylation score. This shows that a high lactylation score frequently suggests a higher degree of malignancy from the perspective of clinicopathological features. We also constructed the nomogram (**Figure 4L**), in which we can classify risks of the patients and predict patient survival by statistically scoring the clinicopathological characteristics and lactylation scores. The results show that if the total score of the patients reached 448 points, the 1-, 2-, and 3-year mortality rates of the patients were 28%, 53.6%, and 66%, respectively. We also draweed the ROC curve of the nomogram (**Figure 4M**). The area of AUCs at 1, 2, and 3 years were all greater than 0.65.

### Immune cells infiltration statement and functional enrichment analysis

**Figure 5:**
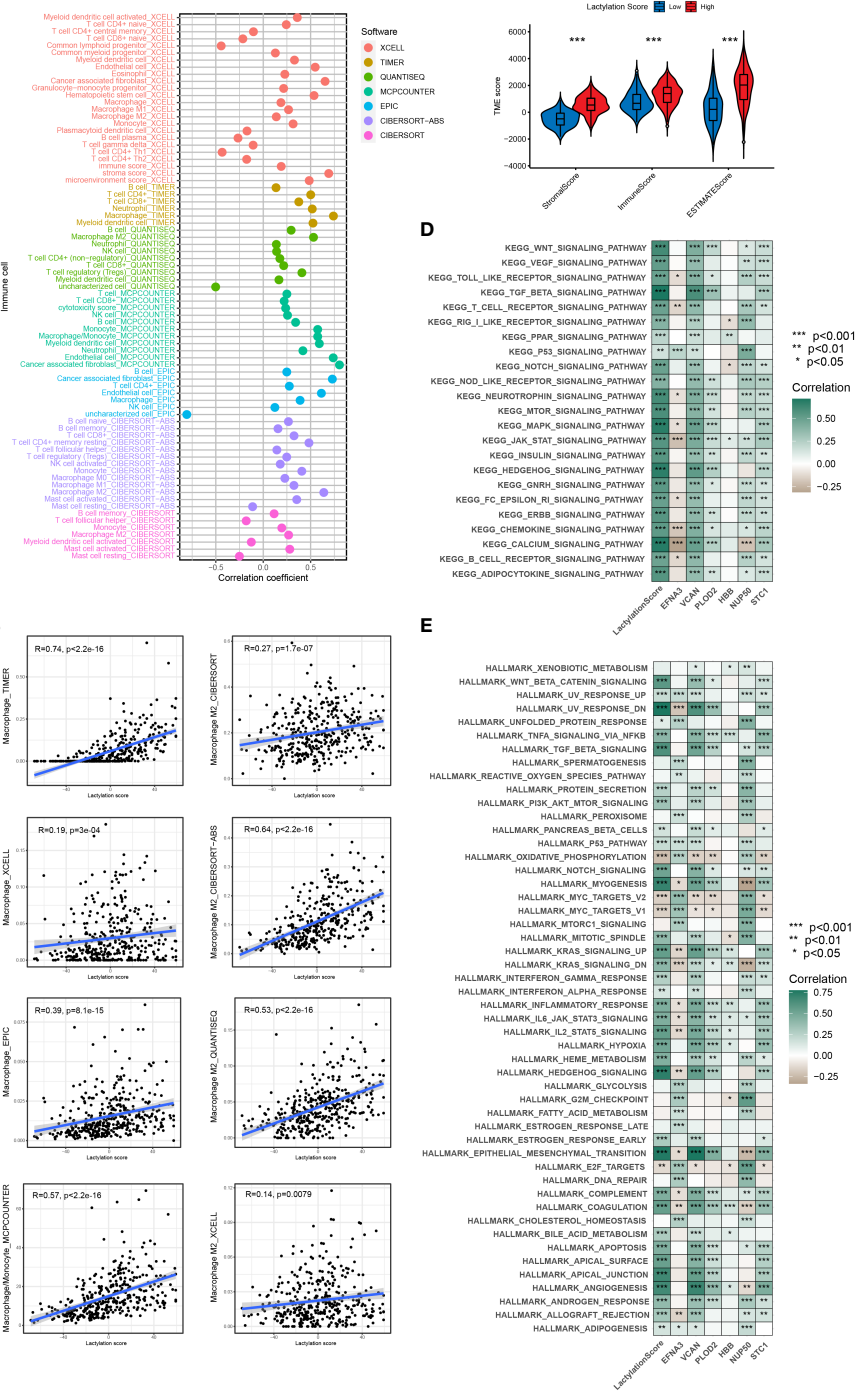
Immune cells infiltration and function enrichment analysis. *p<0.05; **p<0.01; ***p<0.001. **(A, B)** The correlation between lactylation score and immune cell infiltration by various immunocytes analysis methods. **(C)** Correlation between lactylation score and the tumor microenvironment of gastric cancer assessed using the ESTIMATE algorithm. **(D, E)** GSVA analysis of lactylation score and lactylation-related genes.

According to the ESTIMATE algorithm, the stromal and immune scores were statistically higher in high lactylation score group than low lactylation score group. This demonstrated that the group with a high lactylation score had a larger proportion of stromal cell and immune cell infiltration (25). The ESTIMATE score was considerably greater in the group with high lactylation scores than in the group with low lactylation scores, demonstrating a negative correlation between lactylation score level and tumor purity (**Figure 5C**). By combining various immunocytes analysis methods, we analyzed the correlation between lactylation score and immunocytes infiltration (**Figure 5A**). The findings revealed that the degree of macrophage and M2-type macrophage infiltration was positively connected with lactylation score (**Figure 5B**). KEGG and Hallmark enrichment analysis showed that lactylation model was closely relevanted to multiple oncogenic pathways (including WNT, TGF_BETA, MTOR, P53_SIGNALING) (**Figures 5D, E**). At the same time, Hallmark enrichment analysis revealed that the lactylation model was closely relevanted with HALLMARK_EPITHELIAL_MESENCHYMAL_TRANSITION and HALLMARK_ANGIOGENESIS (**Figure 5E**). This indicated that the higher lactylation score, the stronger proliferation, metastasis and invasion ability of gastric cancer.

### Immunotherapy response and immune evasion

**Figure 6:**
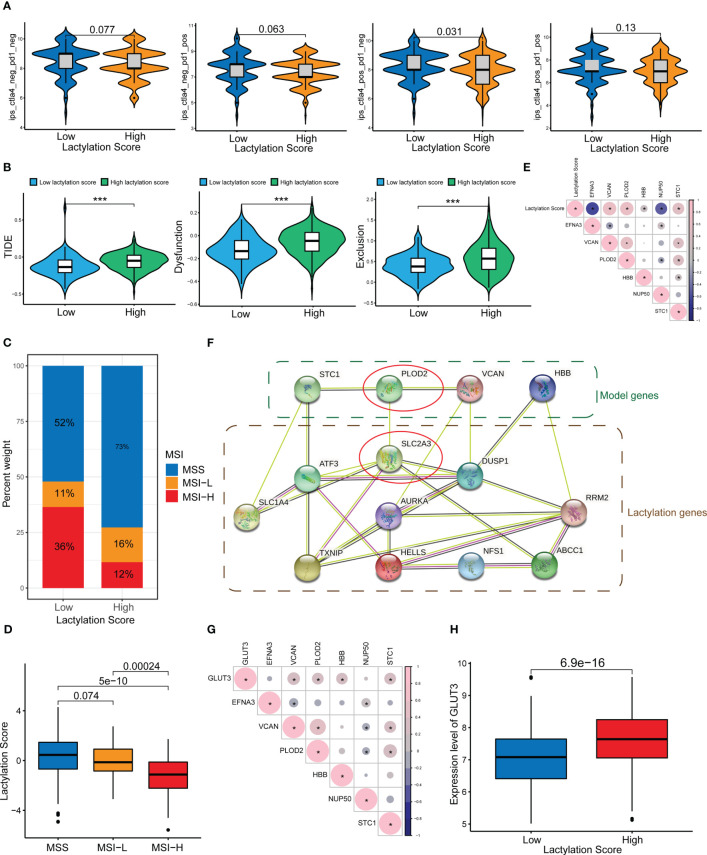
ICIs and immune evasion. *p<0.05; ***p<0.001. **(A)** Sensitivity analysis of the high- and low-lactylation-score groups to immunotherapy. **(B)** TIDE score, Dysfunction score, and Exclusion score of the high- and low-lactylation-score groups. **(C, D)** Microsatellite instability in the high- and low-lactylation-score groups. **(E)** Correlations between the lactylation score and lactylation-related gene expression. **(F)** PPI network between lactylation-related proteins and lactylation target genes. **(G)** Correlation between GLUT3 and lactylation-related genes. **(H)** GLUT3 expression in the high- and low-lactylation-score groups.

Our study also revealed that the lactylation score was relevanted to the efficacy of immune checkpoint treatment. **Figure 6A** shows that patients with lower lactylation scores possessed a higher response rate to ICIs(ctla4_pos_pd1_neg, p<0.05). Additionally, high lactylation score group are more liabled to acquire ctla4 immunotherapy resistance. We also predicted the drug sensitivity of the high- and low-lactylation score groups, and found that most drugs expressed higher sensitivity in the low-lactylation score group, while only two drugs (Gefitinib and Metformin) showed higher sensitivity in the high-lactylation score group (**Figure S1**). Immune evasion can result in resistance to immunotherapy. An algorithm called the TIDE score is used to determine T-cell malfunction and rejection in different tumor types. The scores can be used to forecast the impact of immune checkpoint therapy in patients with tumors in addition to being consistent with immune evasion features (26). **Figure 6B** demonstrates that the high-lactylation-score group’s TIDE, Exclusion, and Dysfunction score were all considerably greater than those in the low-lactylation-score group. Further resulting in immune evasion and immunotherapy resistance was the high-lactylation-score group’s increased susceptibility to immunological dysfunction and immune rejection. We also looked at the lactylation score and the instability of the microsatellites. The low-lactylation-score group showed increased microsatellite instability, as shown in **Figures 6C, D**.

### Expression of lactylation-related genes in GC

**Figure 6E** shows the correlation between the lactylation score and lactylation genes. We constructed a PPI network of lactylation-related genes/proteins and lactylation target genes reported in the literature (**Figure 6F**). At the center of the PPI network, PLOD2 and SLC2A3 (GLUT3) had a greater priority. Additional, there was an interaction between PLOD2 and GLUT3. We speculate that PLOD2 may affect the lactylation level of GC through GLUT3, thereby leading to the development and progression of GC. **Figure 6G** shows the correlation between GLUT3 and lactylation genes, where it can be seen that GLUT3 and PLOD2 showed a strong connection. Moreover, the high-lactylation-score group’s expression level of GLUT3 was noticeably higher than the low-lactylation-score group’s (**Figure 6H**).

### The relation between PLOD2, GLUT3 and lactylation in GC

**Figure 7:**
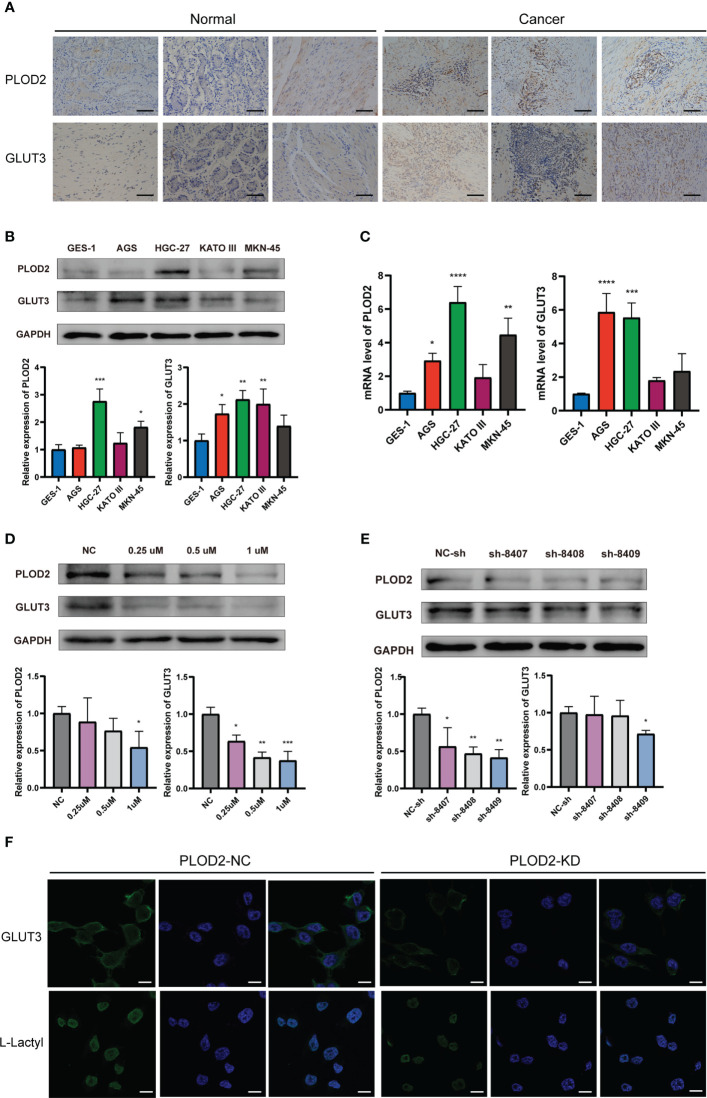
Expression of PLOD2 and GLUT3 in GC cell lines and GC tissues. *p<0.05; **p<0.01; ***p<0.001; ****p<0.0001. **(A)** Immunohistochemistry for PLOD2 and GLUT3 in normal gastric tissues and GC tissues. Scale bar, 100 µm. **(B)** Western blots for PLOD2 and GLUT3. **(C)** PCR results of PLOD2 and GLUT3. **(D)** Western blots of HGC cells treated with different concentrations of lactate dehydrogenase inhibitors (0.25 µM, 0.5 µM, and 1 µM). **(E)** Western blots after transfection with the PLOD2-KD plasmid. **(F)** Expression levels of GLUT3 and L-Lactyl in PLOD-NC group and PLOD-KD group were compared by immunofluorescence. Scale bar, 200 µm.

We performed immunohistochemistry in normal gastric tissues and GC samples (**Figure 7A**). The figure shows that the expression degrees of PLOD2 and GLUT3 in GC samples were all considerably higher than those in normal gastric samples.**Figures 7B, C** shows that PLOD2 and GLUT3 were expressed more highly in GC cell lines than GES-1 cells. The PLOD2 expression was significantly high in HGC-27 and MKN-45, while GLUT3 expression was the highest in HGC-27.

To observe whether PLOD2 and GLUT3 were associated with lactylation, we selected different concentrations of lactate dehydrogenase inhibitors to treat HGC-27 cells for 48 h. **Figure 7D** shows that PLOD2 and GLUT3 were both decreased after HGC-27 were treated by the lactate dehydrogenase inhibitor. When the lactate dehydrogenase concentration was set as 1 µM, the inhibitory effect on PLOD2 and GLUT3 was the most significant. We also constructed three PLOD2-knockdown plasmids expressing heterogeneous nuclear RNAs, namely, sh-8407, sh-8408, and sh-8409. After transfection of each of these three plasmids individually into HGC cells, the expression of PLOD2 was knocked down at the protein level, and the knockdown effect of sh-8409 was the most significant (**Figure 7E**). Therefore, we chose the sh-8409 plasmid to create a PLOD2-KD group of cells. Knockdown of PLOD2 by sh-8409 also downregulated the expression of GLUT3 (**Figure 7E**), further supporting a PPI relationship between PLOD2 and GLUT3. L-Lactyl is a pan-antibody of lactylation modification that reflects the level of lactylation in tissue samples. Immunofluorescence assay results showed that GLUT3 and L-Lactyl expression were significantly declined after PLOD2 knockdown (**Figure 7F**). This indicated that PLOD2 could regulate lactylation modification in GC cell line.

### Functional phenotype of PLOD2 in GC cells

**Figure 8:**
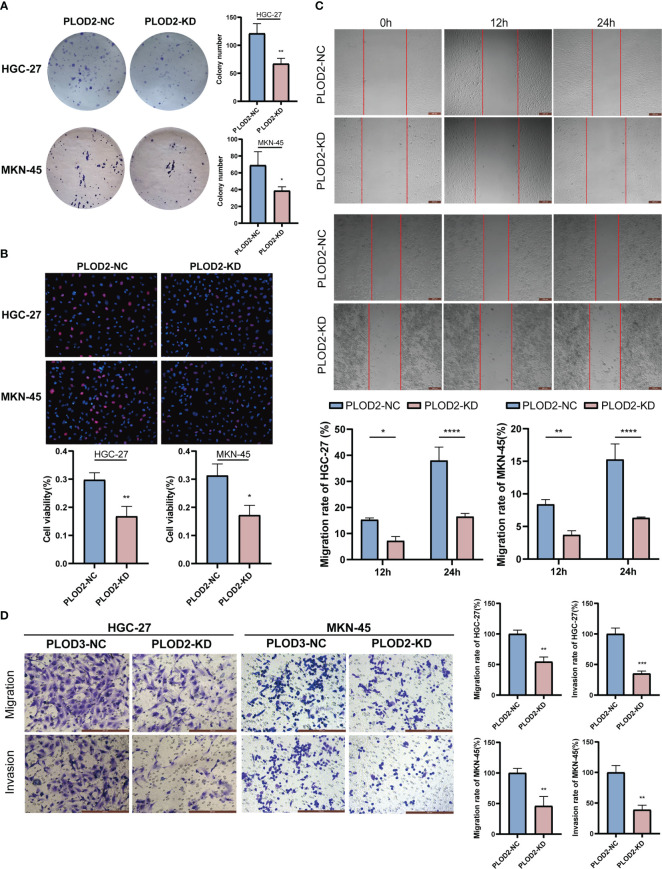
Vitro assay results of PLOD2 in gastric cancer cell.*p<0.05; **p<0.01; ***p<0.001; ****p<0.0001. Scale bar, 200 µm. **(A)** Plate cloning assay results of *PLOD2*-NC and PLOD2-KD in HGC-27 and MKN-45 cells. **(B)** EdU assay results of PLOD2-NC and PLOD2-KD in HGC-27 and MKN-45 cells. **(C)** Wound-healing assay results of PLOD2-NC and PLOD2-KD in HGC-27 cells. **(D)** Transwell assay results of PLOD2-WT and PLOD2-KD in HGC-27 and MKN-45 cells.

After knocking down PLOD2, we examined the functional phenotyoe change in GC cells. **Figure 8A** shows that there were considerably fewer cell clones in PLOD2-KD than in PLOD2-NC. EdU experiment also show that the cell viability of the PLOD2-KD group was significantly reduced (**Figure 8B**). In the scratch assay, the cells in the PLOD2-KD group showed considerably broader scratches than PLOD2-NC group (**Figure 8C**). Similarly, the Transwell assay demonstrated that PLOD2 gene knockdown prevented HGC-27 and MKN45 cells from migrating and invading (**Figure 8D**).

## Discussion

Recently, growing evidence has shown that lactate is not only the most important direct source of nutrition for tumor cells, it can also promote the growth, proliferation, metastasis, drug resistance, and immunosuppression of tumors, such as by acidifying the immune microenvironment and increasing the expression of tumor resistance proteins (27, 28). More importantly, researchers at the University of Chicago demonstrated that lactate is an important epigenetic modification molecule that can affect degree of macrophage polarization through histone lactylation (12). The lactylation modification of histone lysines is indeed widespread in human and mouse cells and is regulated by glycolysis. In addition, the lysine lactylation of histones is highly sensitive to lactate produced by glycolysis, which can change with the intensity of glycolysis or the level of lactate. Protein lactylation modification is the farthest known downstream molecular mechanism of glycolysis, and lactate in the adjustment of cellular functions.

To investigate the function of GC lactylation modification, we downloaded four lactylation pathways with significantly elevated expression in GC tissues from the GSEA database. Six lactylation-related genes correlatived with GC development were screened by cluster analysis, and PCA analysis. A lactylation score model was constructed, which was closely associated with increased tumor infiltrating immunocytes, genetic instability and ICI treatment. The six lactylation-related genes in the constructed model wee also closely correlated with the prognosis of GC.

Tumor growth relies heavily on glycolysis. Tumor cells can produce lactate through aerobic glycolysis and maintain a high-lactate environment, thereby inhibiting T cells that attack tumor cells. Moreover, lactate enhances the expression of regulatory T cells to contribute to the defense of malignant cells, thereby evading the attack of the immune system (29). In this study, the TIDE score of high-lactylation-score group was considerably higher than low-lactylation-score group, which means that the higher lactylation score, tumor cell more likely achieve immune evasion and immune dysfunction. Experiments have shown that the key factor in tumor drug resistance is lactate, and the lactate concentration and glycolysis rate can reflect the sensitivity of tumor drugs to a certain extent (30). Meanwhile, higher glycolysis rate of cancer cells is closely related to lower response rate to ICI treatment (31). We also performed drug sensitivity analysis on the lactylation score model. The findings revealed that high-lactylation-score group’s sensitivity to ctla4 immunotherapy was lower than low-lactylation-score group’s. It proves that patients with high lactylation scores are more likely to develop resistance to immunotherapy, which agrees with the findings of the majority of recent investigations. The above results showed that lactate and lactylation are closely related to immune evasion and sensitivity to ICI treatment. Moreover, lactate and lactylation have received extensive attention as novel target for tumor immunotherapy (32).

LDHA is one of the key enzymes in the reprogramming of glucose metabolism in the TME. It is also the hub protein that connects various cellular metabolic pathways. It directly or indirectly activates signal transduction pathways and regulates immune responses to participate in development and progression of tumors (33). The increase in LDHA level is mainly caused by the increase in tumor glycolytic activity and tumor hypoxic necrosis, which are important drivers of the immunosuppressive microenvironment. LDHA can promote the conversion of pyruvate to lactate, and its activity is positively correlated with the Warburg effect (34). The enzymatic activity of LDHA is regulated by posttranslational modifications, including lactylation, acetylation, and phosphorylation (35). Some studies have shown that when LDH is knocked out, the lactylation level is also inhibited (12). Wang et al. reported that by directly binding to LDHA and PKM2, HULC increases their phosphorylation levels to regulate their activity and ultimately accelerate glycolysis and promote cell proliferation (36). Chen et al. found that CENP-N affects tumor progression by participating in the aerobic glycolysis process of nasopharyngeal carcinoma cells (37). These studies fully demonstrate that aerobic glycolysis and lactylation modification are involved in the regulation of posttranslational modification and that LDHA inhibitors can inhibit the lactylation process by inhibiting the activity of the LDHA enzyme. Current clinical studies are testing antitumor drugs targeting LDHA (38). In our study, after the use of LDHA inhibitors to block lactylation in GC cells, the expression of the lactylation-related gene PLOD2 and the lactylation target gene GLUT3 were inhibited to varying degrees, and the degree of inhibition was positively correlated with the concentration of LDHA inhibitor. This also indicates that the expression levels of the PLOD2 and GLUT3 proteins are regulated by lactylation modification.

PLOD2 is a functional enzyme located in the rough endoplasmic reticulum of the cytoplasm. It participates in the posttranslational modification of collagen and promotes the synthesis of collagen fibers. Fibrotic collagen plays a key role in promoting tumor invasiveness (39). PLOD2 can participate in extracellular matrix remodeling by promoting the deposition of collagen fibers, thereby improving the invasiveness of tumor cells (40–44). In a variety of tumors, clinical data indicate that increased PLOD2 expression can be used as an independent poor-prognostic factor and is associated with poor patient survival (45). This is consistent with the results of our study, in which the overall survival rate of GC patients in the high-PLOD2-expression group was significantly lower than that in the low-PLOD2-expression group, and PLOD2 was the highest-ranked hub gene of the lactylation score model, which was also validated by immunohistochemistry. We found that the expression of PLOD2 in GC tissues, especially in GC foci, was significantly higher than that in normal gastric tissues. At the same time, after PLOD2 knockdown, the proliferation and invasiveness of GC HGC-27 and MKN-45 cells were significantly reduced. TGF-β1 seems to be an important factor in the regulation of PLOD2 (46–48). Through clustering analysis of lactylation-related genes, including PLOD2, we found that cluster 2 GC was enriched in the TGF_BETA_SIGNALING_PATHWAY and ECM_RECEPTOR_INTERACTION pathways, which is consistent with our other results.

The glucose transporter is one of the most important transmembrane proteins in the human body, with a total of 14 subtypes (GLUT1-GLUT14). Among them, GLUT3 encoded by the SLC2A family is a tissue-specific glucose transporter with high affinity for glucose in the GLUT family. GLUT3 is highly expressed in tumor cells and promotes the transport and uptake of glucose by tumor cells (49–52). The affinity of GLUT3 for glucose is five times that of the well-known GLUT1 transporter (53). Taekyu Ha et al. reported that Caveolin-1 factor upregulated glucose transport and uptake and aerobic glycolysis by promoting direct binding of HMGA1 to the GLUT3 promoter region (54). Liu reported that the transcriptional repressor family member ZBTB7A could inhibit the transcription and expression of GLUT3, and the knockdown of ZBTB7A could increase glucose transport and uptake as well as the synthesis of lactate (55). Wang et al. demonstrated that the enhancement of the insulin-stimulated PI3K/Akt phosphorylation pathway can promote the expression of GLUT3, LDHA and monocarboxylic acid transporter 1 (MCT1) (56). Through the above studies, we found that GLUT3 is closely related to glycolysis and lactylation. Therefore, we selected *GLUT3* as the lactylation target gene. We found that the expression level of *GLUT3* was increased in GC cell lines and GC tissues. The results show that lactate dehydrogenase inhibitors could affect the expression of GLUT3 by inhibiting the lactylation level.

In this study, by analyzing the lactylation pathways with elevated component expression in GC tissues, we found lactylation-related genes that play a role in the occurrence and development of GC and constructed a lactylation score model. Lactylation score in GC was closely associated with tumor mutational load, genomic instability, response to ICI treatment, immune cell infiltration, and immune evasion. These findings provide novel ideas for the diagnosis and treatment of GC, and lactylation-related genes may become novel tumor markers or therapeutic targets. Although our paper has many strengths, it also has limitations. For example, further studies are needed to reveal the pathways through which lactylation-related genes affect immune cell infiltration and genomic instability in GC. The accuracy of the lactylation score in predicting the response to ICI treatment by GC still needs to be verified by large-scale clinical trials.

In summary, the lactylation score might be useful in the molecular classification of GC, as it could help to identify different patterns of immune infiltration and genomic instability. The lactylation score can also be used as a method to assess the response of patients to ICI treatment.

## Data availability statement

The datasets presented in this study can be found in online repositories. The names of the repository/repositories and accession number(s) can be found within the article/**Supplementary Materials**.

## Ethics statement

The studies involving human participants were reviewed and approved by the Ethics Committee of the second affiliated hospital of Harbin Medical University(sydwgzr2021-161). The patients/participants provided their written informed consent to participate in this study.

## Author contributions

HY: Bioinformatics analysis, manuscript writing, vitro assays. XZ: Conceptual design, manuscript revision. SY: Prepare tissue and conduct Immunohistochemistry, graphical visualization. AZ: Experimental design and supervision. NL: Experimetal design, manuscript editing. ZM: Conceptual design, bioinformatics analysis, manuscript revision. All authors contributed to the article and approved the submitted version.

## Supplementary material

Supplementary Figure 1 Drug sensitivity in the high and low lacylation score groups.
