# Supplementary figures and images for: Identification of lactylation related model to predict prognostic, tumor infiltrating immunocytes and response of immunotherapy in gastric cancer
Source: Front Immunol. 2023 Mar 3;14:1149989. doi: 10.3389/fimmu.2023.1149989 (PMC10020516; doi:10.3389/fimmu.2023.1149989)

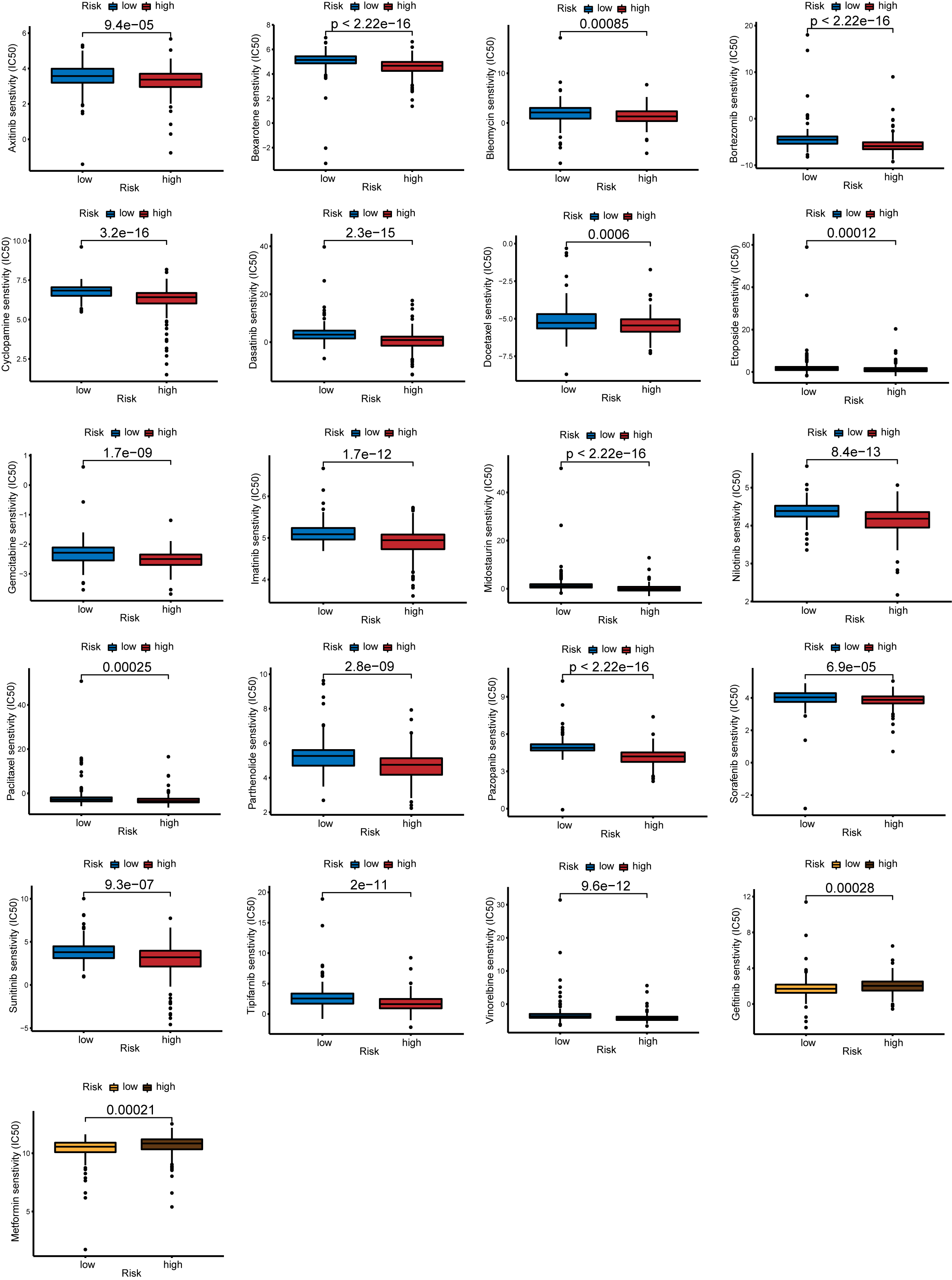

Supplement: Supplementary Figure 1 — Drug sensitivity in the high and low lacylation score groups. [file Image_1.tif]
